# Multi-Access Edge Computing (MEC) Based on MIMO: A Survey

**DOI:** 10.3390/s23083883

**Published:** 2023-04-11

**Authors:** Mengyu Zhu, Shaoshuai Gao, Guofang Tu, Deyuan Chen

**Affiliations:** School of Electronic, Electrical and Communication Engineering, University of Chinese Academy of Sciences, Beijing 101408, China

**Keywords:** multiple input multiple output, multi-access edge computing

## Abstract

With the rapid development of wireless communication technology and the emergence of intelligent applications, higher requirements have been put forward for data communication and computing capacity. Multi-access edge computing (MEC) can handle highly demanding applications by users by sinking the services and computing capabilities of the cloud to the edge of the cell. Meanwhile, the multiple input multiple output (MIMO) technology based on large-scale antenna arrays can achieve an order-of-magnitude improvement in system capacity. The introduction of MIMO into MEC takes full advantage of the energy and spectral efficiency of MIMO technology, providing a new computing paradigm for time-sensitive applications. In parallel, it can accommodate more users and cope with the inevitable trend of continuous data traffic explosion. In this paper, the state-of-the-art research status in this field is investigated, summarized and analyzed. Specifically, we first summarize a multi-base station cooperative mMIMO-MEC model that can easily be expanded to adapt to different MIMO-MEC application scenarios. Subsequently, we comprehensively analyze the current works, compare them to each other and summarize them, mainly from four aspects: research scenarios, application scenarios, evaluation indicators and research issues, and research algorithms. Finally, some open research challenges are identified and discussed, and these indicate the direction for future research on MIMO-MEC.

## 1. Introduction

In recent years, with the arrival of the 5G era, the Internet of Everything has gradually become a reality, and some smart mobile applications have emerged, such as augmented reality (AR) and virtual reality (VR). Some future concepts such as metaverse and digital twin have also been proposed. All of these novel applications suggest that data traffic will continue to explode. According to Ericsson, global mobile data traffic will increase fivefold by 2025 compared with 2019. As a result, these applications with intensive computing requirements demand higher transmission rates, which will put more stringent requirements on mobile communication [1,2].

The emerging mobile applications are usually computationally intensive and delay-sensitive, and put a heavy burden on traditional cloud computing, thus mobile edge computing (MEC) has attracted a lot of attention as a new computing paradigm. The concept of MEC was first proposed by the European Telecommunication Standards Institute (ETSI) in 2014 [3]. While in 2016, ETSI extended the access method from cellular network to other access methods such as WLAN, that is, the concept of mobile edge computing was extended to multi-access edge computing. Unlike cloud computing, MEC aims to provide a distributed service environment by deploying servers closer to mobile users, which can enable low latency and high speed access, alleviating the heavy data burden of cloud computing backhaul [2,4]. In the Internet of Everything era, mobile devices’ access volume and data traffic are at the stage of explosive growth. Thus, MEC has attracted the attention of researchers due to its great superiority in reducing system delay, saving energy consumption, improving service quality, enhancing physical security and increasing cache efficiency. Many researchers have proved that MEC can effectively alleviate the situation of the insufficient computing capacity and limited power of mobile devices in the fields of Internet of Things (IoT) [5], and it is considered as one of the most promising technologies for 5G and beyond networks [6].

On the other hand, the explosive growth of data volume puts forward higher requirements for the capacity of communication systems; thus, another research hotspot in the field of communication is multiple input multiple output (MIMO). Since the earliest experimental system of MIMO was proposed by researchers in the Bell laboratory in the 19th century, it has received widespread attention from researchers all over the world. In addition, in terms of its ability to accommodate plenty of users at the same time, the advantages of effectively improving the spectral efficiency (SE) and the capacity of the system have been proven [7], while with the explosive growth of global traffic data [8], researchers are no longer satisfied with the performance improvement brought by MIMO, and have instead focused on large-scale MIMO systems [9], which require more antennas to be deployed at the base station (BS). Massive MIMO (mMIMO) technology can effectively utilize space resources to cope with the current severe situation of spectrum resource shortage. In addition, mMIMO can provide diversity gain and effectively suppress interference, which has a significant effect on improving the stability and reliability of the system, and its advantage also lies in the improvement of energy efficiency (EE) [10]. When the number of antennas is sufficient, the interference between users is significantly reduced, and the channel can be regarded as orthogonal. Moreover, the mMIMO takes full advantage of the number of antennas to achieve an increase in communication freedom degree [11,12].

At the beginning, MEC mainly focused on the related research about single-antenna systems, but researchers noticed that the huge gain brought by MIMO technology in terms of SE effectively promoted offloading in MEC. Moreover, the MIMO system has evolved from a concept to a practical application step by step and has already been integrated into advanced wireless network standards, making it possible for it to be applied to MEC networks [13]. Therefore, some researchers have tried to conduct research in the scenario of deploying multiple antennas at the BS. As far as we know, ref. [14] took the lead in introducing MIMO technology into the MEC system, and then more and more researchers became involved.

By deploying abundant antennas at the BS, MIMO-MEC can accommodate more users, and multiple users can offload data simultaneously, which can effectively reduce queuing delay. It can also significantly reduce the delay caused by wireless transmission of data, further reducing the overall delay of the entire MEC system [15]. In addition, when the number of antennas equipped at the BS is large enough, the channels of each user are approximately orthogonal, i.e., a single BS can provide basically interference-free signals to the user terminals within its coverage area [11]. Similarly, when a large number of antennas are deployed on the BS in the mMIMO system, the dimension of the channel matrix tends to be infinite. In this case, the randomness of the relevant parameters in the channel matrix is reduced to deterministic expression and concentrated on the diagonal elements of the channel matrix, which is called “channel hardening” [16,17]. This weakens the impact of the slow change in channel characteristics, improves the robustness of communication and has a great advantage over traditional MEC networks, such as the systems based on Time Division Multiple Access (TDMA) and Frequency Division Multiple Access (FDMA), etc.

Researchers focused on MEC and made a breakthrough, and there are many review papers to summarize these results, which are mainly about the architecture, computing offloading, resource management, resource provisioning, service migration and advantages [6,18,19,20,21]. Ming Zeng et al. introduced mMIMO-assisted MEC in [22], but only a brief analysis of the relevant work prior to 2020 was conducted. The authors of [22] focused on demonstrating the advantages of MIMO-MEC through experimentally based numerical analysis. However, considering that the MIMO-MEC network is a promising technology for the next generation communication and studies on the mMIMO-assisted MEC network have kept appearing in the past two years, a comprehensive review and analysis of MIMO-MEC is essential. The lack of attention given to MIMO-MEC in existing reviews has driven us to investigate systematically. We categorize studies from the perspective of research scenarios, techniques, indicators, research issues and optimization methods to provide researchers with a comprehensive and up-to-date view.

In this review, we adopt the three-layer MEC architecture that is popular and widely accepted by many works. It contains the thing layer, edge layer and cloud layer. Offloading strategy and resource allocation in MEC can be operated among users, the edge and cloud to make full use of existing computing resources. 

Compared with the single BS scenario, multiple BSs can cooperate with each other and realize the maximum utilization of resources through joint management, which has a broader application prospect. Thus, the multi-BS cooperative mMIMO-MEC models are introduced in Section 2, in which communication between BSs is considered to achieve mutual cooperation. The common system indicators are also summarized. These models and indicators are the foundation for further investigation in the MIMO-MEC field.

Additionally, we present a state-of-the-art review concerning the field of mMIMO-MEC. There are four main areas of interest, including research scenarios, application scenarios, evaluation indicators and research issues, and research algorithms, which are covered in detail in Section 3. Finally, we discuss the prospects for possible future research directions and open research challenges in the field of mMIMO-MEC in Section 4.

In order to facilitate the readers’ understanding, the most commonly used acronyms in this paper are listed in Acronym and Notation.

## 2. Basic Models

In this section, we introduce the basic models in mMIMO-MEC networks. After analyzing several studies from the literature, the multi-BS cooperative mMIMO-MEC models are summarized, including the task model, communication model and computing model. The BSs are managed by Software Defined Networking (SDN) technology and they can communicate with each other to support collaboration and service migration. However, users can only communicate with one BS. These models are not only a summary of existing research, but can also be well adapted to future research because more and more researchers are focusing on the advantages of cell collaboration, which is of guiding significance. 

At present, the most widely popular and used MEC network is three-layer architecture, including the thing layer, edge layer and cloud layer. The thing layer is the layer where intelligent terminal devices reside, which usually generate a large amount of complex data. The edge layer is the core of the three-layer structure, equipped with the edge servers, which can not only process data but also manage resources. The cloud is undoubtedly the most powerful data processing and storage center, connected to the edge layer through the core network but at a distance from the users. Subsequent studies were carried out on the basis of this three-layer structure.

**Figure 1:**
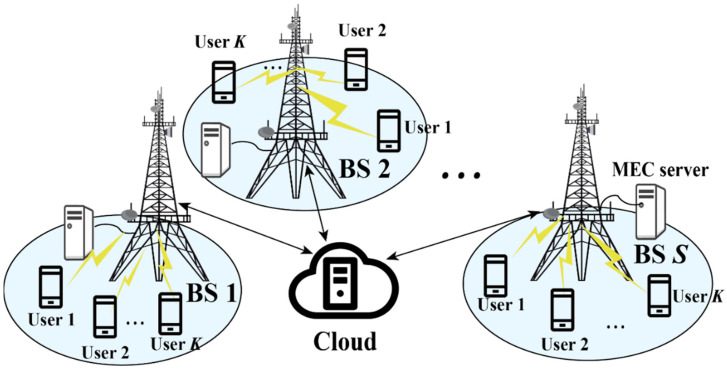
Basic MIMO-MEC system model.

As shown in Figure 1, the mMIMO-MEC network model includes a cloud server, *L* BSs connected to *L* MEC servers, and *K* single antenna users exist in the service scope of each MEC server. Each BS is equipped with *M* antennas as access points (APs), and the MEC servers have communication, computing, storage and other service functions. This section considers the typical mMIMO system where M≫K. In the subsequent parts of this survey, the BS, AP and MEC server are referred to as the same thing and may be used alternately. All the user terminals have a single computationally intensive task requirement, and the computing task can be calculated locally, by the MEC server or by the cloud server under the premise of considering the local computing capacity, energy consumption limitation and latency limitation. The indexes of the MEC servers and mobile users are represented as l∈ℒ≜{1,…,L} and k∈K≜{1,…,K}, respectively. Data offloading is carried out over wireless channels between the user terminal and BS, while the BSs are wired to the cloud. 

The main parameters used in this paper are summarized in Acronym and Notation for readability.

### 2.1. Task Model

This paper considers a frequently used task model as follows. The computing task of each user is defined as a 4-tuple set $T_{lk}=\left\{\lambda_{lk},x_{lk},\tau_{lk},\alpha_{lk}\right\}$. $\lambda_{lk}$ is the size of the input data (bits) for a computing task $T_{lk}$. $x_{lk}$ denotes the number of central processing unit (CPU) cycles required to process per input data bit (cycles/bit). $\tau_{lk}$ represents the maximum tolerable delay for completing each task (s), and we assume that the delay budget does not exceed one time slot, i.e.,$\tau_{lk}\leq \tau$. $\alpha_{lk}$ stands for the ratio of the output data size to the input data size. Thus, the number of CPU instruction cycles required for calculating task $T_{lk}$ is $\lambda_{lk}x_{lk}$, and the size of the calculation result that needs to be transmitted is $\lambda_{lk}\alpha_{lk}$. Note that $\lambda_{lk},x_{lk},\tau_{lk},\alpha_{lk}$ are assumed to be known because they are easily obtained by a task profiler [23].

### 2.2. Computing Models

This subsection introduces a computing model for cloud–edge–things collaboration, including delay and energy consumption under different conditions. 

To make the model more general, it is assumed that the user’s computing task can be jointly completed by the user and the cloud server, or the user and the MEC. Accordingly, the task of the user terminal can be divided into two parts. The offloading ratio is recorded as ϑ, indicating that data of size $\vartheta \lambda_{lk}$ is offloaded to the cloud server or MEC servers, and the rest $(1-\vartheta )\lambda_{lk}$ is performed locally.

#### 2.2.1. Local Computing

The local computing capability of users can complete part of the computing task. Let $f_{lk}$ denote the computing capacity of the user terminal, i.e., the number of CPU cycles, and thus, the local processing delay is calculated by $t_{loc}=(1-\vartheta )\;\lambda_{lk}x_{lk}/f_{lk}$. In this process, the energy consumed by the user itself is $E_{loc}=\kappa_{loc}f_{lk}^{2}(1-\vartheta )\lambda_{lk}x_{lk}$, where $\kappa_{loc}$ is an effective switching capacitance constant that depends on the power coefficient of the chip architecture. 

#### 2.2.2. Computation Offloading

The offloading part can be performed by the MEC server or the cloud according to the actual situation. When the computing task is offloaded to BS, a binary decision $a_{k\to l}$ is introduced for indication, and $a_{k\to l}=1$ means that the BS *l* will complete the calculation of the offloading part. The computing delay is $t_{lk,m}=\vartheta \lambda_{lk}x_{lk}/f_{k}^{l}$, where $f_{k}^{l}$ denotes the CPU cycle frequency (cycles/s) allocated by the MEC server *l* to the user *k*, which is constrained by the total computing capacity of the MEC servers:

(1) $$\sum_{k=1}^{K}a_{k\to l}f_{k}^{l}\leq f^{M},\;\forall l\in ℒ$$

The corresponding energy consumption is $E_{lk,m}=\kappa_{s}f_{k}^{l\;2}\vartheta \lambda_{lk}x_{lk}$, where $\kappa_{s}$ stands for the hardware dependence constant of the MEC server.

Introduce a new binary variable $a_{coo}$ and $a_{coo}=1$ indicates that the BS collaboration mechanism is enabled. The handover data $\lambda_{coo}\leq \vartheta \lambda_{lk}$, which can be controlled by SDN. When $a_{coo}=1$, the computing delay includes two parts: $t_{coo1}=(\vartheta \lambda_{lk}-\lambda_{coo})x_{lk}/f_{k}^{l}$ and $t_{coo2}=\lambda_{coo}x_{lk}/f_{coo}$, where $f_{coo}$ is allocated by SDN and satisfies the computing capacity constraint. The corresponding energy consumption is $E_{coo}=\kappa_{s}f_{k}^{l\;2}\left(\vartheta \lambda_{lk}-\lambda_{coo}\right)x_{lk}+\kappa_{s}f_{coo}^{2}\lambda_{coo}x_{lk}$.

Introduce another new binary variable $a_{k\to c}$. If it is calculated by the cloud server, $a_{k\to c}=1$, and the calculation delay is $t_{lk,c}=\vartheta \lambda_{lk}x_{lk}/f_{c}$. The computing rate of the cloud is much higher than that of the MEC server, so its computing rate $f_{c}$ is considered as a constant for any computing task. In most cases, the energy consumption of the cloud server due to computing is ignored; however, ref. [24] considers the computing cost that is proportional to the size of task data. Thus, it is expressed as $E_{lk,c}=\kappa_{c}\vartheta \lambda_{lk}$, where $\kappa_{c}$ is the cost factor in J/bit.

In some studies, it is assumed that the users’ computing tasks are not separable, i.e., binary offloading decision. In this case, only one of the user terminals, the MEC server and the cloud server can be selected to carry out the task calculation, and the above models can match the binary decision by adjusting the offloading ratio ϑ to 0 or 1.

### 2.3. Communication Model

The whole process of computing offloading includes uplink data transmission, computation and downlink result transmission. The communication model of the MIMO-MEC network is based on multi-user MIMO, whose uplink channel is the multiple access channel and downlink channel is the broadcast channel. The work about mMIMO technology in the communication model section benefits from the derivation and summary of Marzetta et al. [25].

It is assumed that the channel fading remains unchanged over a time slot τ, and the channel matrix from the user to the MEC server is defined as $G_{s}^{l}\in {\mathbb{C}}^{M\times K}$. The channel gain is expressed as $g_{sk}^{lm}=h_{sk}^{lm}\sqrt{\beta_{sk}^{l}}$, where $h_{sk}^{lm}$ represents the small-scale fading coefficient between the user *k* in MEC server *s* and the *m*th antenna in MEC server *l*, and $\beta_{sk}^{l}$ denotes the large-scale fading coefficient between the user *k* in MEC server *s* and the BS *l*. Moreover, the data transmission rate is independent of small-scale fading due to channel hardening [11,25]. The channel models considered in different studies are different, and the detailed analysis is shown in Table 1.

#### 2.3.1. Channel Estimation

Generally, since the channel conditions are unknown or variant during the information transmission, pilot estimation is required. At each BS, every mobile user sends a pilot sequence with the length of $\tau_{p}$ at first, then the BS uses the Minimum Mean Square Error (MMSE) method to estimate the channel. The target cell *l* is called the home cell. All the cells that use the same pilot sequence as the home cell are recorded as ${𝒫}_{l}$, which causes pilot contamination because of the reuse of the pilot [26]. The MMSE estimate is:

(2) $${\hat{g}}_{sk}^{lm}=\frac{\sqrt{\tau_{P}p_{{}_{sk}}^{P}}\beta_{sk}^{l}}{\sigma^{2}+\tau_{P}\sum_{l^{\prime }\in {𝒫}_{l}}p_{{}_{l^{\prime }k}}^{P}\beta_{l^{\prime }k}^{l}}[\sqrt{\tau_{P}p_{{}_{sk}}^{P}}\sum_{s\in {𝒫}_{l}}G_{s}^{l}+W_{Pl}{]}_{\mathrm{mk}},\;s\in {𝒫}_{l}$$

where $p_{sk}^{p}$ is the transmitting power when the user sends the pilot, and $W_{pl}\sim CN\left(0,I\sigma^{2}\right)$ denotes the additive noise at the BS. 

The mean square channel estimate obtained by the BS is given by:

(3) $$\gamma_{sk}^{l}=E\left\{{\left|{\hat{g}}_{sk}^{lm}\right|}^{2}\right\}=\frac{\tau_{P}p_{{}_{sk}}^{P}{\left(\beta_{sk}^{l}\right)}^{2}}{\sigma^{2}+\tau_{P}\sum_{l^{\prime }\in {𝒫}_{l}}p_{{}_{l^{\prime }k}}^{P}\beta_{l^{\prime }k}^{l}}$$

The channel transmission performance largely depends on channel estimation quality, and an effective channel estimation method can significantly reduce the bit error rate [27].

**Table 1:** Comparison of channel models in MIMO-MEC.

Paper	Channel Model	Large-Scale Fading	Small-Scale Fading
[8]	Quasi-static block fading	Pathloss model (LoS): $L_{m,n}\left(\mathrm{dB}\right)=20\log{\left(\frac{4\pi f_{c}D_{n,m}}{c}\right)}^{a}+\xi_{LoS}{}^{b}$	-
[15]	-	Pathloss model: $\mathrm{PL}\;\left(\mathrm{dB}\right)=30.6+36.7log_{10}\left(d\right)$	Rayleigh fading
[28]	Flat fading model	Pathloss and shadow	-
[29]	-	3GPP pathloss model: $\mathrm{PL}\;\left(\mathrm{dB}\right)=148.1+37.6log_{10}\left(d\right)$	Rayleigh fading
[30]	Gaussian–Markov block fading autoregressive model	-	Rayleigh fading
[31]	-	Log-normal shadowing model	Fading coefficient
[32]	Block fading model	3GPP Urban Microcell model: $\beta_{lk}\left(\mathrm{dB}\right)=-30.5-36.7log_{10}\left(\frac{d_{lk}}{1\;m}\right)+SF_{lk}{}^{c}$	-
[33]	Saleh–Valenzuela model	-	-
[34]	Block fading	Pathloss and shadow: $\beta_{lk}=L_{mk}^{-\alpha }{}^{d}\cdot 10^{\frac{Z_{mk}{}^{e}}{10}}$	Rayleigh fading

^a^ $D_{n,m}$ represents Euclidean distance, c stands for speed of light and $f_{c}$ represents carrier frequency. ^b^ $\xi_{LoS}$ stands for average additional loss of free space propagation loss. ^c^ $SF_{lk}$ represents log-normal shadow fading. ^d^ $L_{mk}$ means distance between the kth UE and mth AP (in km), α stands for path loss exponent. ^e^ $Z_{mk}$ stands for shadow fading.

#### 2.3.2. Uplink Multiple Access Channel

After channel estimation, the user terminal offloads the data to the BS. It is assumed that the signals sent by the terminals meet the requirements that the mean value is zero, the power is 1 and they are not related to each other. 

Generally, the receiver adopts Maximum Ratio Combining (MRC) technology [28,35] or Zero Forcing (ZF) technology [15,30] for signal detection. Different signal detection methods can obtain different decoding rates. The uplink achievable transmission rate for the user *k* in BS *l* is given as

(4) $$r_{u,lk}=b_{lk}log_{2}\left(1+{\mathrm{SIN}R}_{lk}^{\mathrm{ul}}\right)$$

where $b_{lk}$ is the bandwidth of the user *k* in BS *l*. If the MRC detection is used,

(5) $$SINR_{lk}^{ul}=\frac{M\gamma_{lk}^{l}p_{lk}}{\sigma^{2}+\sum_{s\in {𝒫}_{l}}\sum_{u=1}^{K}\beta_{su}^{l}p_{su}+\sum_{s\notin {𝒫}_{l}}\sum_{u=1}^{K}\beta_{su}^{l}p_{su}+M\sum_{s\in {𝒫}_{l}\backslash \{l\}}\gamma_{sk}^{l}p_{sk}}$$

where $p_{lk}$ is the transmitting power of the user *k* in BS *l*. The items in the denominator represent the noise interference at the receiver, the non-coherent interference from the pilot contaminating cells, the non-coherent interference from the non-contaminating cells and the coherent interference from the contaminating cells, and if ZF technology is used, then

(6) $$SINR_{lk}^{ul}=\frac{(M-K)\gamma_{lk}^{l}p_{lk}}{\sigma^{2}+\sum_{s\in {𝒫}_{l}}\sum_{u=1}^{K}(\beta_{su}^{l}-\gamma_{su}^{l})p_{su}+\sigma_{r}^{2}}$$

(7) $$\sigma_{r}^{2}=\sum_{s\notin {𝒫}_{l}}\sum_{u=1}^{K}\beta_{su}^{l}p_{su}+(M-K)\sum_{s\in {𝒫}_{l}\backslash \{l\}}\gamma_{sk}^{l}p_{sk}$$

Therefore, the uplink transmission delay of the user *k* in BS *l* is $t_{lk,utr}=\frac{\vartheta \lambda_{lk}}{r_{u,lk}}$, and the energy consumption is $E_{lk,utr}=p_{lk}t_{lk,utr}$. If the power control is considered, the users’ transmitting power should be multiplied by a corresponding power factor.

#### 2.3.3. Downlink Broadcast Channel

For downlink transmission, in the current studies, Time Division Duplex (TDD) operation is mostly used [36,37,38], thus the channel matrix of the downlink broadcast channel can be defined by wireless channel reciprocity as $H=G^{T}$, without downlink channel estimation.

The BS *l* first preprocesses the signals of *K* users with an *M* × *K* precoding matrix $A_{l}$ in the downlink. The precoding matrix $A_{l}$ only depends on the channel estimation within the corresponding BS and is normalized. The form of the downlink achievable transmission rate for the user *k* in BS *l* is $r_{d,lk}=b_{lk}log_{2}\left(1+{\mathrm{SIN}R}_{lk}^{dl}\right)$, which is similar to (4). For MRC and ZF technology, the signal-to-noise ratios are shown in (8) and (9), (10), respectively:

(8) $$SINR_{lk}^{dl}=\frac{M\gamma_{lk}^{l}p_{l}\eta_{lk}}{\sigma_{lk}^{2}+\sum_{s\in {𝒫}_{l}}\sum_{u=1}^{K}\beta_{lk}^{s}p_{s}\eta_{su}+\sum_{s\notin {𝒫}_{l}}\sum_{u=1}^{K}\beta_{lk}^{s}p_{s}\eta_{su}+M\sum_{s\in {𝒫}_{l}\backslash \{l\}}\gamma_{lk}^{s}p_{s}\eta_{sk}}$$

(9) $$SINR_{lk}^{dl}=\frac{(M-K)\gamma_{lk}^{l}p_{l}\eta_{lk}}{\sigma_{lk}{}^{2}+\sum_{s\in {𝒫}_{l}}\sum_{u=1}^{K}(\beta_{lk}^{s}-\gamma_{lk}^{s})p_{s}\eta_{su}+\sigma_{s}^{2}}$$

(10) $$\sigma_{s}^{2}=\sum_{s\notin {𝒫}_{l}}\sum_{u=1}^{K}\beta_{lk}^{s}p_{s}\eta_{su}+(M-K)\sum_{s\in {𝒫}_{l}\backslash \{l\}}\gamma_{lk}^{s}p_{s}\eta_{sk}$$

where $p_{s}$ denotes the total transmission power of BS s, $\sigma_{lk}$ denotes the noise power at the user and $\eta_{lk}$ denotes the power allocation coefficient assigned to the user *k* in BS *l*, satisfying the following condition:

(11) $$\sum_{k=1}^{K}\eta_{lk}\leq 1,\;\forall l\in ℒ$$

Thus, the downlink transmission delay of the user *k* in BS *l* is $t_{lk,dtr}=\frac{\vartheta \alpha_{lk}\lambda_{lk}}{r_{d,lk}}$, and the corresponding energy consumption is $E_{lk,dtr}=P_{l}\eta_{lk}t_{dtr}$.

#### 2.3.4. Communication between BS and Cloud

There is a wired connection between BS and the cloud server, and the transmission rate can be regarded as a constant. The delay and energy consumption of data transmission from BS to the cloud server are $t_{lk,umc}=\frac{\vartheta \lambda_{lk}}{r_{mc}}$ and $E_{lk,umc}=t_{lk,umc}p_{l}$. The delay and energy consumption of the downlink from the cloud to the BS are $t_{lk,dmc}=\frac{\vartheta \alpha_{lk}\lambda_{lk}}{r_{mc}}$ and $E_{lk,dmc}$=$t_{lk,dmc}p_{c}$, respectively, where $r_{mc}$ represents the transmission rate between the MEC server and the cloud, and $p_{c}$ denotes the transmitting power of the cloud server. 

#### 2.3.5. Communication between BSs

BSs are interconnected by capacity-limited wireless links, and the transmission rate between BSs can be regarded as constant [39], which is represented by $r_{mm}$. The delay and energy consumption of data transmission between BSs are $t_{lk,umm}=\frac{\lambda_{coo}}{r_{mm}}$ and $E_{lk,umm}=t_{lk,umm}p_{l}$, respectively. The delay and energy consumption of the downlink are $t_{lk,dmm}=\frac{\alpha_{lk}\lambda_{coo}}{r_{mm}}$ and $E_{lk,dmm}$=$t_{lk,dmm}p_{l}$, respectively.

### 2.4. Summary of Optimization Indicators

#### 2.4.1. Delay and Energy Consumption

The total delay and total energy consumption of the whole system are summarized as follows.

If there is coordination between the BSs, the total delay for the cooperative calculation part is determined by the maximum delay of the two processes: $t_{lk,coo}=\max\left(t_{coo1},t_{coo2}+t_{lk,umm}+t_{lk,dmm}\right)$.

Thus, if the offloading part of the calculation task is completed by the BS, the offloading delay and energy consumption of the user *k* in BS *l* are:

(12) $$t_{lk,off}=t_{lk,utr}+t_{lk,dtr}+\left(1-a_{coo}\right)t_{lk,m}+a_{coo}t_{lk,coo}+t_{pro},$$

(13) $$E_{lk,off}=E_{lk,utr}+E_{lk,dtr}+a_{coo}E_{lk,m}+a_{coo}\left(E_{lk,umm}+E_{lk,coo}+E_{lk,dmm}\right)$$

where $t_{pro}$ represents the processing delay, which includes the extraction of effective information, error correction and other processes required by the BS after receiving the data, as well as the acquisition of required auxiliary information from the cloud server [40]. However, the processing delay is often closely related to the performance and cache content of the edge server, and it has usually been neglected in most existing studies. 

Otherwise, the offloading part will be completed by the cloud server. Then the delay and energy consumption of the user *k* in BS *l* are:

(14) $$t_{lk,off}=t_{lk,utr}+t_{lk,umc}+t_{lk,c}+t_{lk,dmc}+t_{lk,dtr},$$

(15) $$E_{lk,off}=E_{lk,utr}+E_{lk,umc}+E_{lk,c}+E_{lk,dmc}+E_{lk,dtr}.$$

Thus, the total delay and energy consumption of the system are shown in (16) and (17), respectively.

(16) $$t=\sum_{l=1}^{L}\sum_{k=1}^{K}\max\{t_{lk,loc},\;t_{lk,off}\}$$

(17) $$E=\sum_{l=1}^{L}\sum_{k=1}^{K}\left(E_{lk,loc}+E_{lk,off}\right)$$

#### 2.4.2. Spectral Efficiency and Energy Efficiency

In addition to the most commonly used indicators above, the SE and EE can be used as key optimization indicators for MIMO-MEC networks. SE is defined as the system throughput per unit of bandwidth [41]; the SEs of the uplink and downlink are expressed in (18), where the total bandwidth is calculated under ideal conditions. However, the actual bandwidth may be lower since multiple cells may use the same frequency and there will be interference between the cells. If the system bandwidth is fixed, the SE can also be directly expressed as the sum access rate. EE is defined as the ratio between the sum reachable rate and the aggregation of the emitted power [42], and the uplink and downlink EEs are shown in (19).

(18) $$\eta_{\mathrm{SE},ul}=\frac{\sum_{l=1}^{L}\sum_{k=1}^{K}r_{u,lk}}{\sum_{l=1}^{L}\sum_{k=1}^{K}b_{lk}},\;\eta_{\mathrm{SE},dl}=\frac{\sum_{l=1}^{L}\sum_{k=1}^{K}r_{d,lk}}{\sum_{l=1}^{L}\sum_{k=1}^{K}b_{lk}}$$

(19) $$\eta_{EE,ul}=\frac{\sum_{l=1}^{L}\sum_{u=1}^{K}r_{u,lk}}{\sum_{l=1}^{L}\sum_{u=1}^{K}p_{lk}},\;\eta_{EE,dl}=\frac{\sum_{l=1}^{L}\sum_{u=1}^{K}r_{d,lk}}{\sum_{l=1}^{L}p_{l}^{\prime }}$$

where the transmitting power $p_{lk}$ can be adjusted by dynamic voltage and frequency scaling, and $p_{l}^{\prime }$ represents the actual power used by the BS. Usually, the two indicators need to be considered jointly, and a compromise optimization is carried out to save spectrum resources and transmission energy to the greatest extent. 

## 3. Research Status

In this part, as many studies as possible on MIMO-MEC are collected. Furthermore, we summarize the research content and analyze the current research status from four main aspects, i.e., research scenarios, application scenarios, evaluation indicators and research issues, and research algorithms. 

### 3.1. Research Scenario

**Figure 2:**
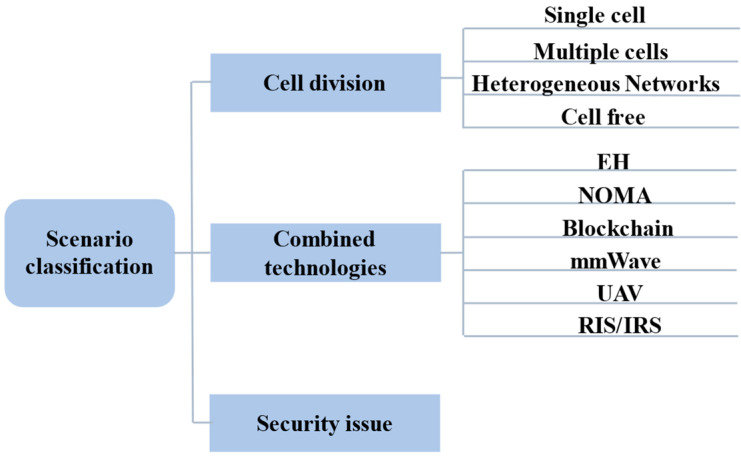
Research scenario classification.

The research of MIMO-MEC is usually carried out in different cell setting scenarios, and the combination with different technologies is explored to meet various needs. Moreover, security is a major concern for researchers, thus, the studies in the literature are classified using the above three aspects, and the classification of the research scenario is summarized in Figure 2. 

#### 3.1.1. Cell Division

##### Single Cell

**Figure 3:**
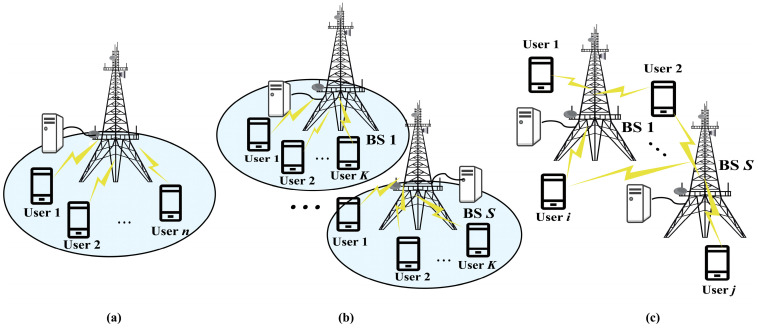
Different cell division scenarios: (**a**) single cell, (**b**) multiple cells and (**c**) cell-free.

Because MIMO technology can give full play to its advantages in the case of multiple users, researchers usually assume a scenario with a large amount of users who request access simultaneously, which is regarded as the research background. In fact, the number of BSs also has an impact on the offloading effect in edge computing, and when mMIMO technology is applied to MEC, the pilot contamination and signal interference between BSs cannot be ignored. Some studies start with simple scenarios and are carried out in the single BS scenario with less interference, which is shown in Figure 3a.

In [15], it is assumed that all the computing tasks must be offloaded to the BS for calculation, and ZF technology is applied to suppress the interference between users at the BS under perfect and imperfect channel conditions, respectively. The scenario set in [28,35] is almost the same as that in [15], but it is only studied under the condition of an imperfect channel state. In [43], the Full Duplex (FD) BS is used for *K* downlink users’ data transmission and *J* downlink mobile devices’ task offloading. It is attempted to reduce downlink transmission power by using Multi-User Interference (MUI) of the downlink instead of suppression. Compared with the studies mentioned above, refs. [29,44] also take into account the users’ local computing capacity, and adaptively choose to calculate locally or completely offload the tasks to the BS. Different from binary offloading, partial offloading is another offload strategy, i.e., the computing task can be arbitrarily split into two parts and completed by the local and BS at the same time, which is more flexible. It is assumed in [30] that the task arrives randomly and the channel condition is time-varying, and the offloading ratio is used to indicate how much data need to be offloaded. Reference [45] also assumes that tasks arrive randomly but queue according to the order they arrive, with each mobile device maintaining a queue. However, the above studies all assume that the calculation results of the tasks are very few and directly ignore the downlink transmission. In contrast, Nguyen et al. in [46] consider the transmission of calculation results. Their studies were carried out under perfect and imperfect channel conditions, respectively, and subsequently, the research was extended by taking the downlink channel into consideration, which is more realistic, although it leads to a more complex optimization problem.

##### Multiple Cells

Some researchers go a step further and consider a more complex multi-cell scenario, which is illustrated in Figure 3b. Although the multiple cell scenario brings an improvement in the offloading efficiency, it also causes some problems such as intensified interference. In the actual scene, there is inter-cell interference caused by pilot contamination, and the interference grows along with the number of antennas [47]. 

In [3], Sardellitti et al. set up a multi-cell static scene, and the user terminals were also deployed with multiple antennas. The computing task can be completed jointly by the local and the cloud server, while the BSs in this paper have no computing capacity and only act as a transmission relay. According to the collected information such as user status, channel status and other information, the cloud server divides users into two subsets in advance. Some user terminals need to offload for calculating, while others only need communication resources. Furthermore, a more realistic scenario is considered by R. Malik and M. Vu [31]. Without knowing the complete Channel State Information (CSI), the MRC detection and precoding are used to complete the uplink and downlink transmission, and the calculation task is partially offloaded to the BS, while in [48], it is assumed that each BS serves the same number of users. The users and the BSs are equipped with multiple antennas, and the users occupy the same frequency in different cells, which may cause mutual interference. 

We notice that the multi-cell scenario not only expands the coverage of a BS but also improves the QoS of users with the help of BS cooperation. Reference [31] studies the task offloading of edge users in collaborative MEC scenarios. The system contains two BSs with overlapping areas, and in this cooperative transmission area, BSs fully collaborate and share both CSI and transmission data, enabling the cell–edge devices to communicate with two BSs simultaneously, which can give full play to the role of collaborative BSs.

##### Heterogeneous Networks

Theoretically, Heterogeneous Networks (HetNets) are based on multi-cell scenarios, but compared with the traditional network, mMIMO HetNets are further improved and more suitable for practical scenarios. Usually, it is studied in a scenario where there exists a multi-antenna macro station and multiple single-antenna small BSs simultaneously to serve multiple users. According to this model, ref. [49] provides a spectrum and energy efficiency evaluation framework for the network. MIMO-MEC is further combined with HetNets to conduct research under the same scene setting, and the offloading decision is assumed to be known [50]. In [51], a framework of MEC HetNets is proposed to reduce the interference and improve system capacity. The network uses mMIMO technology and densely deploys low-power small cells for transmission, including a macro BS connected to the central server. C. Wang et al. demonstrate that mMIMO can improve the data transmission rate, and MEC can facilitate mMIMO transmission in return, complementing each other, and then, storage and fast content retrieval are performed locally. In their proposed framework, it is proved that CSI can be aggregated on the centralized MEC server, i.e., the macro BS. Additionally, the intra-cell and inter-cell interference could be reduced by the cooperative precoding among small cells. By comprehensively utilizing the computing resources and cache resources in the edge computing network, an intelligent cache scheme is proposed to reduce the delay and signaling overhead caused by repeated retrieval of the same content. It is worth mentioning that [51] is one of the very few studies that takes into account cache resource in MIMO-MEC.

##### Cell-Free

As the deployment of BSs becomes more and more intensive, traditional cell divisions lead to frequent cell switching. To ensure that the cells can provide continuous services for all users, the cells are no longer specifically divided. This is the concept of the cell-free (CF) mMIMO proposed by the Bell laboratory [52], and the scenario is shown in Figure 3c. It is a trend to use high-frequency signals to transmit information; nevertheless, the higher the frequency used in a BS for communication, the smaller its coverage range will be. The CF mMIMO networks can better adapt to the development trend of future communication as well as the mobility of users. Through Coordinated Multi-Point Joint Transmission (COMP-JT), the huge power gain is maintained and the problem of the poor signal of edge users is alleviated [53]. It is also proved that the system performance is greatly improved, especially in terms of system throughput and communication quality [54,55,56]. Studies have focused on the CF mMIMO-MEC system, in which all users within the coverage range of the BS can establish connections with it. 

Various task types of users with different average delay requirements are considered and the users can choose whether to use the MEC server or the central server (cloud) to complete the calculation [32,57,58]. After analyzing the Signal Interference Ratio (SIR) of the uplink and downlink transmission in the interference-restricted channel by using random geometry and queuing theory, the probability of successful communication and successful calculation are derived, and then, the Successful Edge Calculation Probability (SECP) under the target computation delay is given. The influence of the BS coverage rate and the central server offloading probability on SECP, and the influence of SECP on total system energy consumption are further analyzed [57]. 

The authors also evaluate the performance of the same system mainly from the perspective of mathematical derivation, but assume that if the task is completed by MEC, the MEC server with the lowest calculation delay is assigned to perform the calculation [58]. Based on the M/M/1 queuing system, the closed expression of communication interruption probability is derived, and the expression of Successful Computation Probability (SCP) under target computation delay is also derived. It announces that for the fixed expected SCP, the EE of the system increases with the decrease in computation delay. Based on the assumption that the access point has hardware damage, the authors of [34] derive the closed expression of SE and EE in the CF mMIMO-MEC network. Finally, the analysis proves that the CF mMIMO system has certain resilience to hardware damage, indicating that the hardware cost of CF mMIMO can be effectively reduced, which lays a theoretical foundation for its application in practice.

Some research scenes in the literature are summarized in Table 2, which is convenient for intuitive comparison and analysis.

#### 3.1.2. Combined with Other Technologies

MIMO-MEC is easy to expand and can also be combined with other technologies to promote each other, so as to further improve system performance. The researchers in [59] have taken Energy Harvesting (EH) into account in the MIMO-MEC system, and study the MIMO-MEC system that can collect energy from the external environment instead of traditional energy supply. However, this paper only studies the very simple scenario of a single BS and single user.

NOMA is also a promising technology for 6G. In [60], the power domain multiplexing technology of NOMA is used to superimpose and encode user information, thus further improving the transmission rate. Hybrid NOMA is a combination of the advantages of NOMA and OMA to balance complexity and performance. Reference [61] combines hybrid NOMA with MIMO-MEC and finds that it can achieve better delay performance than NOMA under energy constraints. It provides a new way to combine NOMA with MIMO-MEC.

Blockchain technology can be regarded as a distributed public ledger, which is used to directly record the transaction information between any nodes in a point-to-point network without the need of a third party. Due to its traceability, and decentralization characteristics, it has become a key research target for researchers. MIMO-MEC can be applied to blockchain networks to significantly increase system throughput and allow more mobile devices to gain access. A MIMO-MEC-assisted blockchain network where devices can offload intensive mining tasks to BSs and store block data in the cloud server is investigated in [62]. 

Millimeter Wave (mmWave) technology is one of the alternative technologies for next generation communication. Although propagation losses are severe at mmWave frequencies, shorter wavelengths make it possible to compress more antenna units into the same physical space, and the narrowband and high-gain beam provided by a large antenna array is used to make up for the deficiency of the propagation channel [42,63]. Combining mmWave with MIMO and then introducing the combination to a MEC network can make full use of space resources, provide larger channel capacity and further improve SE. In [33], MIMO technology is applied to the downlink in multi-BS and multi-user scenarios, and studied in the millimeter band, while a Media Access Control (MAC) protocol called LSMWN-MAC for the downlink mmWave MIMO-MEC system is proposed in [64], which is expected to become the communication paradigm of the MIMO-MEC network. The protocol uses SDMA/FDMA for interaction and data transmission between the AP and users to adapt to the characteristics of mmWave and mMIMO, and accommodate more mobile terminals at the same time. Then, aiming at the situation that the global optimization cannot be achieved due to the change in channel state, an improved protocol (MLSMWN-MAC protocol) is proposed. The application scope of the two protocols is analyzed and compared with 802.11ad. The results show that the proposed protocols significantly improve saturation throughput, which lays the groundwork for the subsequent implementation of a MIMO-assisted MEC network.

Reconfigurable Intelligent Surface (RIS) or Intelligent Reflecting Surface (IRS) is considered to be an innovative technology for 6G, which consists of an array of passive or active reflecting elements. It can create a controlled radio environment by intelligently adjusting the phase-shifts of reflecting elements to achieve the reconstruction and enhancement of wireless propagation. Reference [65] uses EH to charge mobile users, and IRS technology is used to assist the MEC network to improve EH efficiency and the data transmission rate between users and BSs, while IRS and UAV are combined in [66], and a 3D wireless channel model based on controllable MIMO-UAV/IRS is established, which can bypass the blockage of Line of Sight (LoS) and avoid service starvation for users at the edge of the cell.

#### 3.1.3. Security Issue

The security of the MIMO-MEC system is also a hot research direction. Due to the broadcast characteristics of a wireless channel, there is a risk of eavesdropping during the offloading process. Although a multi-antenna system has certain advantages in preventing eavesdropping, the security problem cannot be ignored. 

Zhao et al., in [67], focus on improving the security performance of the MIMO-MEC system, and study how to allocate resources to reduce information leakage and ensure the security of communication under the dual threat of eavesdropping and interference attacked by a malicious user. In the scenario of a single BS and multiple users, there is a malicious eavesdropper who sends strong interference signals. The users’ tasks are offloaded to the MEC server for calculation, and the downlink transmission delay is ignored. In the literature, the uplink security rate is derived, and under the delay constraint, the expression of the actual energy consumption is also derived combining with the security rate of the physical layer. On the basis of the familiar research on resource allocation, the authors add an extra secure offloading constraint, i.e., the offloading rate of each user cannot exceed the secret rate. They jointly optimize the offloading data bits, transmission power and offloading rate on this premise. When solving the problem, the original problem is decomposed. Firstly, the transmission rate is fixed to obtain a convex subproblem, and the closed expressions of offloading data bits and offloading time of uplink are derived by the Lagrange multiplier method. Then, based on the derived formula, the iterative method is used to optimize the transmission power and the maximum offloading rate.

MIMO-MEC, combined with Cognitive Radio (CR) technology, can alleviate the increasing demand for spectrum and computing resources. Reference [68] establishes a CR-based MIMO-MEC environment research scenario against intelligent attacks, including multiple BSs, a primary user, a secondary user and an intelligent attacker, all equipped with multiple antennas, and conduct research without CSI. In the CR scenario, the secondary user’s spectrum resources are restricted by the primary user’s license. The user’s task can be performed jointly by local and idle MEC. An intelligent attacker could choose one from four methods, silence, spoofing, jamming and eavesdropping, to carry out a communication attack. By optimizing the offloading decision, and distributing the transmitting power and the offloading rate, energy consumption, delay and data security transmission are kept in balance. Two resource allocation strategies based on DRL are proposed, which are the Edge Server Selection strategy based on Dyna architecture and Prioritized sweeping (DPESS), and the Edge Server Selection strategy based on Deep Q-network (DESS). The convolutional neural network is trained by using an empirical replay technique and Stochastic Gradient Descent (SGD).

In a smart grid system, security is also considered [69]. There are multiple smart meters in a single cell, as well as a multi-antenna malicious eavesdropper, which can steal the offloading data of some users’ smart meter, or even log into their own servers to perform calculations. The task of the smart electricity meter is completely offloaded to MEC for calculation, and the physical layer security technology is used to prevent eavesdropping and realize the secure communication of the smart grid. It is assumed that the BS and the malicious user know all the CSI between the user and the BS. The user’s achievable safe transmission rate is derived, and then the optimal computational frequency can be obtained. The offloading rate and transmission power are jointly optimized to minimize energy consumption, and the sequential iterative optimization algorithm is used to solve the problem. The results show that if the scheme can be applied to a practical smart grid, i.e., when serving more users, it can save a considerable amount of energy.

Reference [70] studies the collaborative MEC network combined with NOMA. With the help of the helpers at the cell center, the edge user who cannot establish a direct transmission link with the BS can offload the task to the MEC server. The helpers help compute part of the computing task by partial offloading. In order to avoid information leakage to the eavesdropper, PLS technology is used to ensure that the channel gain of the helper is greater than that of the eavesdropper, so as to realize the safe transmission of the cooperative MEC network.

### 3.2. Application Scenarios

**Figure 4:**
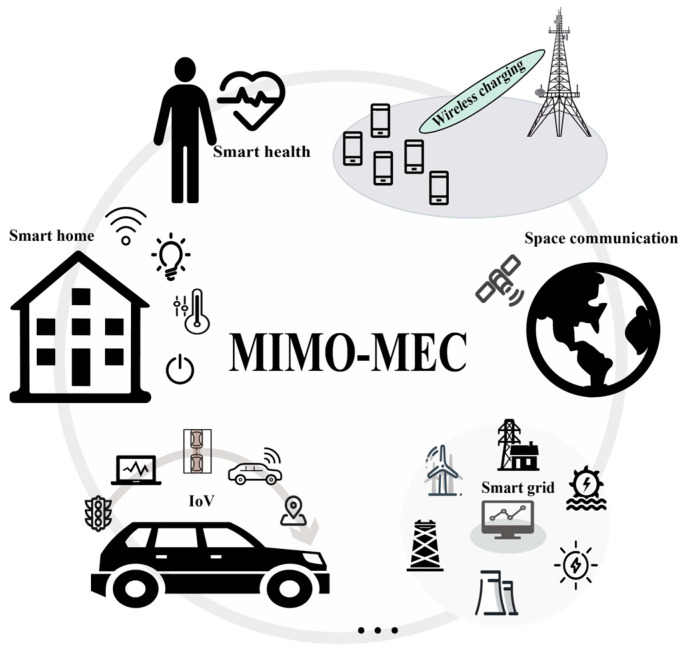
Some application scenarios of MIMO-MEC.

Thanks to the rapid development of IoT technology, tremendous opportunities have been created in many fields, and innovative applications have made it possible to improve the quality of life. Among them, MIMO-MEC also plays an irreplaceable role and is applied in many scenes [71,72]; some scenarios, including smart health and smart home, are illustrated in Figure 4.

#### 3.2.1. Internet of Vehicles

The Internet of Vehicles (IoV) is an important component of the IoT, and MIMO-MEC can also be applied to the IoV to realize the application with strict requirements on calculation, delay and throughput under the condition of limited energy, which is of great significance for the development of electric vehicles. Saving energy consumption can enable electric vehicles to travel more kilometers, which is of primary importance. It is usually recommended to offload all or part of the intensive computing tasks to the MEC server. However, the vehicle network is highly dynamic, so the randomness is greatly enhanced, which has a great impact on the offloading performance. Aiming at 5G technology, in [73], a platform is designed to evaluate the communication performance level of mobile terminals, a BS and other nodes in the central intelligent transportation system. Throughput, delay, network capacity, transmission rate and reliability are taken as the evaluation indexes so that the network performance of mobile terminals (vehicles) in the system can be evaluated.

Unmanned Aerial Vehicle (UAV) technology is often used in IoV networks, and is especially suitable for dealing with emergencies. However, UAV technology is very sensitive to energy consumption and expects a higher transmission rate with lower power. The application of UAV technology in MEC can further shorten transmission distance and increase channel gain [74,75]. In some studies, multiple UAVs are deployed within the range of a single BS, but they only assist users to offload tasks to the ground BS or the cloud for computing as the aerial transmission relay [8,76]. The authors of [77] take the lead in studying the LoS-mMIMO-UAV-assisted MEC network, applying it to the IoV network, and updating the network architecture. A three-dimensional dual MEC network model is proposed, in which one MEC is a Road Side Unit (RSU) with a parallel MEC server deployed on the ground, and the other is an aerial RSU, i.e., UAV. The aerial RSU not only has a MEC server, but it can also be used as an air relay between vehicles and the ground RSU to decode and forward part of the offloading tasks so as to solve the problem that the vehicles cannot communicate directly with the ground RSU due to congestion. In addition, the multi-stage MEC calculation offloading protocol and download protocol are proposed in this architecture. This paper also proposes the concept of three-sided LoS mMIMO and it is assumed that vehicles, the aerial RSU and the ground RSU all adopt two-dimensional (2-D) Uniform Rectangular Planar Arrays (URPA) with a large number of antenna units. 

#### 3.2.2. Wireless Charging

The MIMO-MEC network can not only complete the offloading calculation of users’ computing tasks but also realize wireless charging without physical connection via the wireless power transmission technology. The mMIMO technology can achieve the concentration of energy in a particular direction through beamforming and charge multiple users simultaneously with multiple antennas, which will provide MIMO-MEC with greater application prospects. It may be an effective solution for mobile devices that continuously process the applications requiring heavy computing resources but with limited battery power. Wang et al. in [36] mainly study the Wireless Sensor Network (WSN) assisted by MEC with wireless transmission function. The BSs are equipped with multiple antennas and computing tasks of mobile devices can be calculated locally or offloaded to the cloud through uplink channels, while through the downlink channel, the mobile devices obtain energy and receive information alternately. In each time slot, the user first obtains energy and then only uses this part of energy to complete the task calculation, without consuming the power of the device itself. Reference [37] also studies the scenario where multiple BSs can provide computing offloading and on-demand wireless charging services for multiple users through the downlink. However, the offloading and charging processes cannot be carried out simultaneously. The single BS, multi-user and FD system is studied in [78], and the task is completed jointly by the user and the BS. The energy obtained from the BS is used to complete the local calculation. 

The above studies verified the possibility of the co-existence of offloading and wireless charging in MIMO-MEC networks, opening up a new idea for research into the MIMO-MEC network.

#### 3.2.3. Space Communication

Fu et al. in [79] also apply MIMO-MEC to the satellite IoT system, and use the satellite link to provide communication services for AP. In recent years, Low Earth Orbit (LEO) satellites have also been used in the IoT, which makes the coverage wider and the infrastructure cost lower [80,81]. Its advantage is that even if the ground infrastructure is damaged, satellites can still provide communication services [82]. Although the optical fiber link is replaced by a satellite-AP link to meet the real-world requirements, the scenario settings of [78,79] are basically the same.

#### 3.2.4. Smart Grid

A smart grid can realize information collection and real-time monitoring by deploying a large number of sensors and advanced metering infrastructure but also generates a large amount of data. The introduction of MIMO-MEC can effectively manage resources and save costs. Based on the MIMO-NOMA-MEC system model, the distribution network faults are detected, and part of the information collected by intelligent devices is offloaded to the MEC server for comprehensive analysis and quick troubleshooting so that the system can achieve stable operation [60]. Reference [83] notes the huge energy consumption of the BS, and proposes to equip the BS with renewable energy devices. Combining MIMO-MEC with a smart grid, and utilizing renewable energy generated by the grid assists the BS through two-way energy interaction to maximize the benefits.

### 3.3. Optimization Indicator and Research Issue

#### 3.3.1. Optimization Indicator

The optimization objectives of most studies are about time delay and energy consumption, but there are differences according to the emphasis of research content. 

Some studies only concentrate on delay, for example, Li et al. in [60] aim at minimizing the total latency of all users. Zeng et al. in [15] also consider delay only but minimize the delay of the user who has the largest delay among all users to ensure the communication quality of all users. Huang et al. in [26] and Feng et al. in [35] set the same objective function as [15] under energy consumption and computing resource constraints to ensure the fairness among users, while other studies only consider energy consumption, including [14,48,50,67,78]. The five papers mentioned above only regard minimizing the total energy consumption of users as the goal.

Even though some of the studies are motivated from the energy consumption perspective, the optimization metrics are not identical. Nguyen et al. in [46] aim to minimize the maximum weighted energy consumption of mobile users and achieve fairness among them as much as possible. There are also some researchers who only consider energy consumption, but from the perspective of the whole system. R. Malik and M. Vu aim to minimize the weighted sum of energy consumed by the user and the MEC server under the maximum delay constraint [11,31]. Although MIMO-MEC is applied to IoV, its optimization objective is still to minimize the total weighted energy consumption of mobile users and BSs [77]. However, the mobile users here are vehicles, the UAV is equivalent to the BS and the goal is to reduce power consumption as much as possible so that the service life can be extended.

In fact, both energy consumption and time delay are extremely important for MEC, so researchers usually jointly optimize the two aspects, and the most common objective function is to minimize the weighted sum of system energy consumption and time delay, to balance the QoE and power consumption [8,29,30,43,44,76].

SE and EE are also common criteria for system evaluation. Under the constraints of power budget and delay, the total uplink energy consumption is minimized, while the minimum SE is maximized [32]. In order to ensure fairness among all users, ref. [84] establishes the optimization problem with the goal of maximizing the minimum SE. If the system bandwidth is constant, the offloading rate is equal to SE, which is also a crucial aspect that needs to be considered. The goal of [79] is to maximize the uplink reachable rate, and the weighted sum rate of all users under QoS constraints is maximized in [33]. In [85], maximizing the total computed bits per total energy consumption in the MEC system is regarded as the optimization objective, which is defined as “EE”. In [49], SE and EE are comprehensively considered to maximize their weighted sum while ensuring the minimum QoS requirements of each user.

In general, the optimization of delay and energy consumption is from the perspective of users, giving priority to the user experience, but [83] studies this from the perspective of operators, with the goal of minimizing the transmission power and the required real-time power at the BS.

In fact, in different research scenarios, the objective functions of optimization are not the same due to the combination of different technologies. When EH technology is considered, the battery energy queue needs to be stabilized on the basis of minimizing the time average function of the weighted sum of energy consumption and delay [59]. If MIMO-MEC is to be studied to realize wireless charging, the total energy consumption of the user and BS offloading should be minimized under the restriction of round-trip delay, and the user can obtain the maximum power from charging [37]. Taking wireless charging into consideration as well, ref. [36] sets the goal as the maximization of WSN’s Quality of Service (QoS) so as to improve the system’s data processing capacity per unit period. 

#### 3.3.2. Research Issues

**Figure 5:**
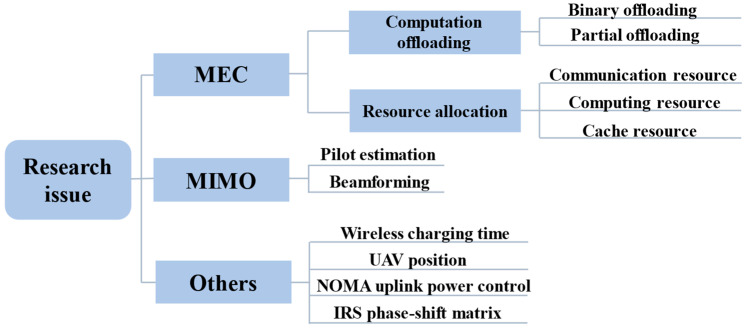
Research issues in MIMO-MEC networks.

The outline of the common research issues in MIMO-MEC networks presented up to now is shown in Figure 5 and described in detail below.

##### Research issues in MEC

Some researches focus more on the MEC network and explore the optimization of the offloading process. References [15,32] jointly optimize the allocation of the uplink transmission power and computing resources. In [30], the local computing power and uplink power allocation for users are optimized.

The offloading strategy and resource allocation are often coupled. Reference [46] also takes the offloading decision into account and jointly optimizes the offloading decision, and computing and radio resource allocation. When partial offloading is used, the offloading decision becomes the offloading ratio. In [31], power control is considered to improve the probability of successful communication. In addition, the offloading ratio, computing resource allocation between user terminal and MEC server, and the duration of different phases are jointly optimized to achieve different optimization objectives so as to improve the system performance. Malik and Vu in [11] optimize the transmission time allocation of the uplink and downlink, computing resource allocation and offloading ratio. The CPU frequency, uplink transmitting power and offloading task size (offloading ratio) are optimized in [50,78,79]. However, ref. [78] gives extra consideration to the uplink rate. The duration of the uplink transmission is considered in [50], while in [79], the ratio of the calculation result to the size of the offloading task is included. 

##### Research Issues in MIMO-MEC

On the basis of MEC optimization, beamforming, pilot estimation and other issues are taken into account to optimize MIMO performance so as to further achieve overall performance improvement. The offloading decision and beamforming are considered in [44]. The uplink transmission power allocation and downlink beamforming vector are optimized in [43], and [49] also takes backhaul bandwidth allocation into account based on [43]. Sardellitti et al. in [14] consider the covariance matrix of MIMO transmitters and the allocation of computational resources, while in [59], it is assumed that the task can be partially offloaded; thus, the optimization of the offloading proportion is considered on the basis of [14]. Moreover, the practical harvested energy and whether to give up the task also need to be optimized in [59]. Reference [29] minimizes the weighted sum of energy consumption and delay by jointly optimizing the offloading decision, multi-user MIMO precoding and computing resource allocation. Reference [33] considers user association, precoding and power allocation. Reference [48] jointly optimizes the communication and computing resources, including precoding, the transmission rate allocation in the uplink and downlink, and the computing resource allocation. References [28,35] emphasize pilot transmission so that pilot transmission delay is added to the total system delay. Under the constraints of energy consumption and computing resources, pilot transmission power, data transmission power and computing resource allocation are jointly optimized to minimize the maximum computational offloading delay among all users. The MIMO-MEC network can give full play to the advantages of MIMO technology, and beamforming is an essential part that needs to be considered in optimization, and has a great influence on the performance of the system [86,87].

##### Research Issues in Other Techniques

However, when considering the combination with different technologies, the focus of the research issues will be different. If wireless charging technology is used, there are additional factors that need to be considered because charging and data transmission in the downlink cannot be carried out at the same time. Malik and Vu in [37] consider the offloading ratio, time allocation for transmission and charging, and beamforming. Similar to those considered in [37], the offloading decision, downlink energy beamforming vector, receiving beamforming vector and dynamic TDD (D-TDD) factor are jointly optimized in [36]. 

If the scene switches to UAV-assisted MIMO-MEC systems, the location of the UAV is quite critical to the transmission rate and power consumption of the system, and usually, the optimization of the three-dimensional position is also taken into account. In a multi-UAV scenario, the offloading decision, UAV position and power allocation are jointly optimized [8,76]. 

If NOMA is considered, then power control must be taken into account [62,88]. If combined with the IRS technique, optimization of the phase-shift matrix is also essential [65,66]. The research objects and issues in the papers that are combined with other technologies are listed in Table 3 for easy comparison.

### 3.4. Optimization Algorithm

Optimization problems in the field of MEC are usually non-convex or NP-hard [18], and optimization models are usually proved to be mixed integer non-linear programming problems [2,44], which are complex and very difficult to solve. The problems in MIMO-MEC are basically the same as those in MEC. In fact, researchers usually carry out combinational optimization on several parts of the offloading decision, computing resource allocation, communication resource allocation, beamforming and pilot estimation, etc. Thus, obtaining the global optimal solution directly is often accompanied with great difficulty and high complexity. As an alternative, suboptimal algorithms with low complexity are proposed.

#### 3.4.1. Convex Optimization

Convex optimization is one of the most widely used methods, and is favored by researchers because of its mature computing system and the ease of obtaining suboptimal results. If the optimization problem can be proved to be convex, the convex optimization method can be used directly, which greatly reduces the difficulty of problem solving. Michailidis et al. in [77] derive the closed solution of transmitting power allocation, time slot scheduling and computing resource allocation through the Lagrange duality method. Then, according to the above results, an optimization algorithm based on the sub-gradient is proposed to solve the problem.

However, in general, the optimization models developed based on MIMO-MEC are usually non-convex. If the optimization model involves multiple optimization objectives, decomposition technology is usually applied to decouple the complex optimization problems into multiple subproblems, such as the offloading decision subproblem, resource allocation subproblem or beamforming subproblem, etc., and then break through one by one. In [20], optimization is carried out under perfect CSI and imperfect CSI conditions, respectively, and the problem is decomposed into three subproblems of beamforming, offloading decision and D-TDD factor optimization, which are solved respectively. In [60], the problem is divided into a task allocation coefficient optimization subproblem and resource allocation subproblem, and a multi-objective iterative algorithm is designed.

The original problem is decoupled into two subproblems in [50,78,79]. The closed solution of the computing resource allocation subproblem is obtained first, and the second subproblem is obtained after substituting this solution, and then, the subproblem is further decoupled. As for the second subproblem, ref. [78] continues to decouple it into three convex subproblems, which are solved by the interior point method. Reference [79] decouples it into two quadratic problems and solves them by an active-set method, while in [50], it is decomposed into a convex subproblem and a non-convex subproblem. The difference of convex functions (DC) method is used to deal with the non-convex structure, and the interior point method is used to solve the problem. The remaining variables are optimized alternately in all three papers.

In fact, after investigating the articles, it is more generally found that the subproblems decoupled from the original problem remain as non-convex or NP-hard. A significant portion of the researches choose to transform them into convex or near-convex problems and then adopt feasible convex optimization methods. After that, the subproblems are solved iteratively until convergence. Reference [46] decouples the original problem into an offloading optimization subproblem and power allocation (PA) subproblem, and under the imperfect CSI condition, a DC method is used to deal with the non-convex structure of the PA subproblem. The distributed local search algorithm is used to solve the user association problem, and then the residual optimization problem is transformed into a rank-constrained D.C. problem, which is solved iteratively [33]. In [44], Quadratic Constrained Quadratic Programming (QCQP) is used to reconstruct the optimization problem, and the semi-definite relaxation (SDR) algorithm is used to solve the offloading decision problem. Based on the optimal offloading decision, the MU-MIMO beamforming design problem is transformed into a convex problem by using the fractional programming method, and then solved by an iterative algorithm. Reference [43] proposes the weighted multi-objective optimization problem in the case of using MUI and suppressing MUI, respectively. Under a perfect CSI condition, it is suggested to solve the beamforming subproblem and PA subproblem by SDR and the Lagrange method, respectively, and the solving process is iterated until the stopping criterion is met. For the imperfect CSI case, convex relaxation and transformation are applied, and the non-convexity of the formula is treated by s-procedure. Considering the lower and upper bounds of the offloading delay in the original problem, ref. [29] uses SDR and the rounding method to obtain the offloading decision. Next, with the offloading decision, fractional programming based on quadratic transformation and the weighted MMSE method is used to realize the MU-MIMO precoding design under two offloading delay conditions.

Successive Convex Approximation (SCA) is one of the most commonly used methods in convex optimization to transform non-convex problems into convex problems [48]. By using the SCA technique, the non-convex problem is transformed into a convex problem and solved by an iterative method under the condition of imperfect CSI [15]. In [14], to begin with, in the case of a single user and single BS, the closed expression of the optimal solution is derived based on the water injection algorithm, and then in the multi-cell scenario, the energy consumption weight is allocated according to the user priority to optimize the weighted sum of all users’ energy consumption. Finally, based on SCA, a centralized algorithm and distributed algorithm are proposed. Reference [49] decouples the original problem and proposes an alternate optimization algorithm combining Lagrange duality and SCA. Using queuing theory and random geometry, an iterative algorithm based on sequential convex programming is used to solve the problem [32]. In each iteration, SCA is used to optimize the correlation convex approximation of the original problem. Lyapunov is a random optimization method that enables online decision making and suboptimal performance [18]. A dynamic computing offloading (DCO) algorithm based on SCA is proposed under the framework of Lyapunov optimization [59].

A double-layer nested algorithm is proposed in [31] to solve the original problem. The outer layer increases monotonously with the offloading proportion, and is solved based on the iterative algorithm. Then the prime-dual algorithm is applied to solve the inner convex problem under the given offloading ratio, in which the dual variables are updated according to the sub-gradient. The closed expressions of the optimal CPU frequency at the user terminal and MEC server derived in this paper are also applied to the prime-dual algorithm to solve the resource allocation subproblem. In another paper [11], the authors of [31] further optimize the algorithm, which still adopts the double-layer nested algorithm, but it is composed of the internal prime-dual algorithm and external delay awareness descent algorithm. The delay awareness descent algorithm adds the modifiable stop criteria to the standard descent method, which can better satisfy the delay constraint. R. Malik and M. Vu continue the idea of a nested algorithm into paper [37]. It determines the solution order according to the importance of subproblems. The offloading problem is solved first, and then turns to the received energy maximization problem. When solving the offloading problem, the nested structure is adopted, and it uses the prime-dual method based on the sub-gradient algorithm internally, and the external algorithm adopts the delay awareness descent algorithm based on the standard Newton method with a modifiable stop criteria, which is similar to [11]. Finally, the optimal offloading proportion and time allocation for offloading are obtained. Based on the result of the time allocation, the two-layer nested algorithm is still adopted to solve the second subproblem. In each iteration, the optimal dual variables of the beamforming direction solution are found through the sub-gradient method externally. Subsequently, the internal algorithm using the standard convex solver is used to solve the linear programming problem and obtain the optimal beamforming power allocation.

#### 3.4.2. Heuristic Algorithm

At present, the heuristic algorithm is one of the most popular methods to solve NP-hard problems. Inspired by nature, the behaviors of animals and other natural creatures are abstracted into algorithms to deal with optimization problems. For instance, a heuristic algorithm based on a genetic algorithm is used to obtain the global optimal solution in [8,76]. In [84], three heuristic algorithms including simulated annealing, differential evolution and particle swarm optimization are used, respectively, to solve the max-min fairness power allocation problem so as to show the superiority of the heuristic algorithm. However, the heuristic algorithm may fall into a local optimal solution, and the algorithm performance is uncertain and easily affected by complex parameters.

#### 3.4.3. Machine Learning

With the development of machine learning technology, advanced artificial intelligence technology has been applied to various fields, and thus, technologies such as deep learning and reinforcement learning have developed rapidly. Moreover, traditional methods usually obtain static solutions of complex optimization problems and cannot make the best decision according to the dynamic environment [18]. In order to overcome the above shortcomings, ref. [30] uses the Deep Reinforcement Learning (DRL) method based on continuous action space, which is summed up as the Depth Deterministic Policy Gradient (DDPG), to learn the decentralized computing offload strategy of all users, and adaptively allocate local computing resources and transmission power according to the local observation of each user.

A temporal attentional deterministic policy gradient based on DDPG is proposed in [45]. They design a temporal feature extraction network including a 1-dimensional convolution residual block and an attentional long short-term memory network to ensure high-quality state representation and function approximation. Moreover, to accelerate the convergence of model training and keep it stable, a rank-based prioritized experience replay method is developed.

#### 3.4.4. Game Theory

Considering the optimization problem as a three-stage Stackelberg game, an iterative algorithm based on backward induction is proposed to realize the Nash equilibrium of the Stackelberg game [62]. The subgame optimization in each stage is analyzed. In the first stage, the optimization of resource service pricing maximizes the profit of cloud service providers. In the second stage, computing capacity and the unit price of the computing service charged by the equipment are optimized to maximize the income of BSs, and the third phase maximizes the profit for a single user by optimizing the computing requirements and block storage policies. The upper bound of traversal throughput and the maximum number of devices connected to the network are also derived. The game theory approach works in a collaborative or non-collaborative way to reach a solution that satisfies all players. The method is flexible and easy to operate. However, it is worth noting that the satisfactory solution may not be the global optimal one.

## 4. Challenges and Future Research Directions

Although the MIMO-MEC network has attracted more and more attention, there are still some constraints in the implementation of mMIMO, such as excessive energy consumption and the high complexity of hardware design. On the one hand, it is difficult to form the full digital beamforming, while the performance of analog beamforming is poor. On the other hand, the unknown CSI makes the implementation of mMIMO more difficult. Despite there being great challenges in research, mMIMO-MEC still has great research prospects, and its future research direction is bound to integrate with other new technologies, adapt to different real scenarios, realize the simultaneous access of many users and continuously improve user communication quality. It will also focus on comprehensive communication integrating ground wireless communication and space satellite communication, with a view to providing high QoS services and finally realizing the interconnection of everything [79,89].

### 4.1. Considering User Mobility

Almost all current studies on MIMO-MEC assume that the users are still or moving slowly, which are static scenarios. However, in the practical application field, the mobility of the user terminal should be taken into consideration. 

User movement produces a Doppler shift related to the speed and carrier frequency, resulting in a decrease in the coherence time. Once the symbol period is greater than the coherence time, fast fading appears. Such a rapidly changing channel greatly reduces the system transmission efficiency and the accuracy of channel estimation. Without using a pilot, ref. [90] designs a blind channel prediction algorithm in a mmWave-MEC scenario to adapt to the fast-changing channel, which provides a good solution.

In addition, considering the mobile users in the multi-BS scenario, it is necessary to consider the selection and handover of the MEC server, and service migration. It can also generate dynamic resource allocation or online resource allocation problems. These problems are particularly important and have become research hotspots in the IoV [91,92]. However, mobility is still an urgent challenge for the MIMO-MEC field in the future.

### 4.2. Channel Estimation and Channel Modeling

In practical applications, the channel conditions are usually unknown. Before offloading the calculation task, the pilot transmission is required for channel estimation, and the quality of channel estimation will directly determine the transmission quality. Therefore, channel estimation is also a very important research issue in MIMO-MEC. 

Since the whole system is delay-sensitive and energy consumption is limited, the energy consumption and delay of the transmitting pilot should also be weighed and optimized. If too much time and energy are allocated to pilot transmission, it will affect the completion of the offloading task. Conversely, the quality of channel estimation will not be guaranteed. Coupled with the real-time change in the channel, it will greatly aggravate the difficulty of research. Estimating the channel in dynamic scenarios by using deep learning may be an appropriate strategy [90]. 

Channel modeling is the cornerstone for studying MIMO technology, so it is also crucial for MIMO-MEC. The Rayleigh fading model and 3GPP standardization model are most commonly used in current studies, but these models cannot portray the complete characteristics of massive MIMO channels, including the near-field effect [93] and the non-stationarity of channels that dynamically change in time and space dimensions [94]. Therefore, more refined channel modeling is necessary.

### 4.3. Combined with Machine Learning

Deep learning and reinforcement learning have been applied to the resource allocation of MEC [95,96]. There is no need to know the prior knowledge of the system, and it can dynamically allocate resources, and its advantages are particularly prominent when the parameters in the research scene change dynamically [97,98]. Federal Learning (FL) is a distributed computing paradigm and a kind of ML; it achieves accuracy at the expense of multiple rounds of communication, uses local data to train the global model and aggregates local updates to the cloud or MEC server, which has been used in OFDM-based MEC [99]. However, in our investigation, heuristic algorithms and convex optimization methods are used most frequently in the research of MIMO-MEC networks, and few people utilize FL, reinforcement learning or other machine learning solutions to optimize the objective function.

Developing a resource allocation optimization algorithm based on deep learning, or proposing a low delay and ultra-reliable deep learning architecture for a MIMO-MEC system can provide a good direction for exploring the effective solution of these kinds of complex problems. However, it usually requires additional online or offline training, and the complexity is very high [76]. The difficulty lies in how to obtain a data source, and how to better train the deep learning algorithm and reduce the computational complexity of the algorithm as much as possible.

### 4.4. Combined with Other New Technologies

There are already some studies combining MIMO-MEC with promising technologies such as NOMA or mmWave communication, but there are still many technologies that need to be explored. Visible light communication is also a candidate technology for 6G, as it offers a new possibility to transmit more data without being limited by scarce spectrum resources. It can also complement radio frequency technologies in ultra-dense networks, providing reliable communications [100]. RIS technology is another research hotspot [101], and the combination of RIS and MIMO-MEC is also a newly emerging research hotspot, which is worth further exploration. However, introducing new technology into MIMO-MEC needs to consider various influencing factors comprehensively, and how to ensure all of the different technologies give full play to their advantages is the difficulty.

### 4.5. Integration of Communication, Sensing and Computation

The 6G wireless communication network will further realize ubiquitous connection. Moreover, mobile terminals need to carry out precise and complex computing tasks in highly dynamic environments. Therefore, the responsibility of edge computing is not only to complete the computing task but also to realize the intelligent terminal’s perception of the environment.

The research directions focused on the integration of communication, sensing and computation include: constructing the integration framework of communication, sensing and computation resource allocation for balancing the perception accuracy, the communication and computing capability [102,103]; and realizing the fusion of communication and sensing signals through the design of joint transmitting of and receiving waveforms [104]. 

## 5. Conclusions

In this survey, we comprehensively review the research status of MIMO-MEC networks. Firstly, multi-BS cooperative mMIMO-MEC models are summarized and cell collaboration is taken into account, which are very easy to expand and suitable for most research backgrounds. These models lay the foundation for a better understanding of relevant research. Secondly, an in-depth overview of the research on MIMO-MEC is presented, from the aspects of research scenarios, application scenarios, evaluation indicators and research issues, and research algorithms, and this is the prominent contribution of this paper. Regarding the research issues, we provide a comprehensive summary of the key issues from the technical point of view. Finally, we clarify the shortcomings of the current research, and highlight some promising research directions and challenges in the field of MIMO-MEC, hoping to promote the further development of MEC. 

## Figures and Tables

**Table 2:** Comparison of papers about research scenarios in MIMO-MEC.

BS Setup	Paper	Collaboration Manner	Offloading Granularity	CSI	Explanation
Single	[15,43]	Edge only	Binary	Perfect/imperfect	Must offload to the BS
	[28,35]	Edge only	Binary	Imperfect	Must offload to the BS
	[29,44]	Things–edge	Binary	Perfect	-
	[46]	Things–edge	Binary	Perfect/imperfect	-
	[30]	Things–edge	Partial	Perfect	-
	[45]	Things–edge	Partial	Perfect	Mobile devices maintain their own queues
Multiple	[14]	Things–cloud	Binary	Perfect	BSs transmit only
	[11]	Things–edge	Partial	Imperfect	-
	[11]	Things–cloud	Binary	Perfect/imperfect	-
	[11]	Things–edge	Partial	Imperfect	-
	[48]	Cloud only	Binary	Perfect	Same number of users in each BS, and BSs transmit only
HetNets	[49]	Edge only	Binary	Perfect	Single multi-antenna micro BS, multiple single-antenna small BSs
	[50]	Things–edge	Partial	Perfect	
CF	[32]	Edge–cloud	Partial	Imperfect	-
	[57,58]	Edge–cloud	Binary	Perfect	Probabilistic offloading decision

**Table 3:** Comparison of Research Objects and Issues Combined with Different Techniques.

Combined Technology	Paper	Objective	Research Issue
EH	[59]	Minimize the time average of the weighted sum of energy consumption and delay while stabilizing the battery energy queue.	(a) Whether to complete the task; (b) transmission covariance matrix; (c) computing capacity allocation; (d) offloading ratio; (e) energy harvesting.
NOMA	[60]	Minimize delay.	(a) Transmission power allocation; (b) offloading ratio; (c) computing capacity allocation.
	[88]	Minimize delay	(a) Power allocation; (b) offloading ratio;
Hybrid NOMA	[61]	Minimize delay	(a) Power allocation in NOMA and OMA, respectively
mmWave	[33]	Maximize the weighted sum rate of all users.	(a) Offloading association decision; (b) precoding design; (c) transmission power allocation.
Wireless charging	[36]	Maximize the total number of computed bits.	(a) Offloading decision; (b) D-TDD factor; (c) downlink energy beamforming vector; (d) receiving beamforming vector.
	[37]	Minimize offloading energy consumption and maximize wireless charging energy.	(a) Offloading ratio; (b) time allocation for offloading and charging; (c) energy beamforming vector.
SWIPT	[78]	Minimize energy consumption.	(a) Computing capacity allocation; (b) transmission power allocation; (c) offloading data size; (d) uplink transmission rate.
	[79]	Maximize the uplink reachable rate.	(a) CPU frequency allocation; (b) ratio of the calculated result to the size of the input data; (c) terminal transmitting power allocation; (d) offloading ratio.
UAV	[8,76]	Minimize the weighted sum of delay and energy consumption.	(a) Association decision; (b) transmission power allocation; (c) UAV position.
	[77]	Minimize the total weighted energy consumption of computation and communication.	(a) Transmission power allocation; (b) offloading ratio; (c) time slot scheduling.
RIS-EH	[65]	Minimize total delay.	(a) EH time; (b) data transmission time; (c) offloading ratio; (d) computation resource allocation; (e) MU detection coefficients; (f) phase-shift matrices.
RIS-UAV	[66]	Maximize ϵ-effective EE	(a) Transmit power vector; (b) UAV trajectory; (c) active transmit beamforming vector; (d) passive reflecting coefficient matrix.

## Data Availability

Not applicable.

## Acronym and Notation

**Acronym**	**Definition**	**Acronym**	**Definition**
*AP*	Access point	*MEC*	Multi-access edge computing or mobile edge computing
*BS*	Base station	*mMIMO*	Massive multiple input multiple output
*CF*	Cell free	*MMSE*	Minimum Mean Square Error
*COMP-JT*	Coordinated Multi-point Joint Transmission	*MRC*	Maximum Ratio Combining
*CPU*	Central processing unit	*mmWave*	Millimeter Wave
*CR*	Cognitive Radio	*NOMA*	Non-orthogonal Multiple Access
*CSI*	Channel State Information	*PA*	Power allocation
*DC*	Difference of convex functions	*QCQP*	Quadratic Constrained Quadratic Programming
*DCO*	Dynamic computing offloading	*QoS*	Quality of Service
*DDPG*	Depth Deterministic Policy Gradient	*RIS*	Reconfigurable Intelligent Surface
*DRL*	Deep Reinforcement Learning	*SCA*	Successive Convex Approximation
*EE*	energy efficiency	*SDN*	Software Defined Networking
*EH*	Energy harvesting	*SDR*	Semi-definite relaxation
*FD*	Full Duplex	*SE*	Spectral efficiency
*FDMA*	Frequency Division Multiple Access	*SECP*	Success Edge Calculation Probability
*HetNets*	Heterogeneous Networks	*TDD*	Time Division Duplex
*IoT*	Internet of Things	*TDMA*	Time Division Multiple Access
*IoV*	Internet of Vehicles	*UAV*	Unmanned Aerial Vehicle
*LoS*	Line of Sight	*WSN*	Wireless Sensor Network
*MAC*	Media Access Control	*ZF*	Zero Forcing
**Notation**	**Description**	**Notation**	**Description**
*L*	Number of BSs	$\kappa_{loc}$	Effective switching capacitance constant
*K*	Number of users in each BS	$\kappa_{s},\;\kappa_{c}$	Cost factor for BS, cloud
*M*	Number of antennas	$a_{k\to l},\;a_{k\to c},$ $a_{coo}$	Indicates whether to offload to BS, cloud and cooperative BS
$\lambda_{lk}$	Input data size	$g_{sk}^{lm},h_{sk}^{lm},\beta_{sk}^{l}$	Channel gain, small-scale fading and large-scale fading coefficient
$x_{lk}$	Number of CPU cycles required to process per input data bit	$\gamma_{sk}^{l}$	Mean square channel estimate
$\tau_{lk}$	Maximum tolerable delay	$\sigma ,\sigma_{lk}$	The noise power of the BS and the user
$\alpha_{lk}$	Ratio of the output data size to the input data size	$b_{lk}$	Bandwidth of the user *k* in BS *l*
ϑ	Offloading ratio	$p_{lk},p_{l},p_{c}$	Transmitted power of user, BS and cloud
$f_{lk},\;f^{M},f_{c}\;$	Computing capacity of the user terminal, BS and cloud	$\eta_{lk}$	Power allocation coefficient in downlink
$f_{k}^{l}$	User *k*’s computing capacity allocated by BS *l*	$r_{mc},r_{mm}$	Rate between BS and cloud, and between BSs
